# Book Review

**Published:** 1914-09

**Authors:** 


**Achondroplasia : its Nature and Cause.** By Dr. Murk Jansen. Pp. 98. London : Baillière, Tindall & Cox. 1912. Price 6s. net.—This admirable study of achondroplasia should be read by all interested in the subject. The features of this disease are held to fall into two main sets, the one due to dwarfing of cartilage bones, and the other to mechanical malformations of the fœtus. It is with the latter that the author is mainly concerned, and he seeks to show that these are due to pressure by the amnion, either from an amnion unduly small in relation to the size of the fœtus, or to an excess of amniotic fluid. The mechanical deformities of achondroplasia he attributes to an infolding of the head and trunk of the fœtus. These causes are at work from the third to the eighth week of fœtal life. The author brings forward a considerable body of evidence in favour of his thesis, which he states in a particularly lucid way, showing a very wide acquaintance with all the facts that bear upon the case. Whatever view may be taken of his proposition, which for its verification requires further observation and experiment, this study contains a large number of original and suggestive observations, and other theories as to the nature of achondroplasia receive adequate notice. There are a number of excellent illustrations, and altogether this little book forms a valuable contribution to an obscure and interesting disease.

**Mental Deficiency (Amentia).** By A. F. Tredgold. Second Edition, Revised and Enlarged. Pp. 491. London: Baillière, Tindall & Cox. 1914. 12s. 6d. net.—This book is already so well known and so widely appreciated that it needs little further commendation. It is probably the best book upon the subject, treating mental deficiency in a wide and comprehensive manner. It is obviously the outcome of large experience, and is marked by sound judgment, lucidity of statement, and care for what is of practical utility. It is written in a clear and attractive style, and whilst of considerable length, it maintains the reader's interest throughout. The Mental Deficiency Act, 1913, will undoubtedly result in an increased demand for knowledge of this subject on the part of the general practitioner, whose opinion will be more frequently sought in this class of cases, and his responsibility in advising upon them be greater. The chapters on mental tests, case taking, which have been added to this edition, and those on diagnosis and treatment, will be especially valuable to him, and he could not have a better guide. A summary is given of the Mental Deficiency Act, 1913. The present issue has been thoroughly revised, is well illustrated, and well deserves our cordial recommendation.

**Diagnosen av Barnetuberkulosen's Kliniske Initialformer.** By H. G. Gade. Pp. 112. Kristiania: Steen. 1914.—Gade discusses in detail the early signs and symptoms of tuberculosis in children, directing his special attention to the earlier changes in the bronchial glands. To this end he has made a very careful study of thoracic conditions present in children who do not show evidences of tuberculosis, and gives a number of radiograms from children of various ages. He lays considerable stress upon the value of radiograms in connection with bronchial gland lesions, and also describes, at considerable length, the signs upon which he places greatest reliance. The volume is worthy of reference, especially as there is a good bibliography.

**The British Journal of Surgery.** Vol. I. 1913–1914.—We gave a hearty welcome to the *British Journal of Surgery* when the first number was published in July, 1913.[1] Now we have the first four numbers before us bound together in one handsome volume. Briefly, we may say that the book is full of valuable contributions to surgical literature from cover to cover. We find the promise given by the first part has been splendidly maintained in the three parts issued since, so that the *Journal*

[1] See *Bristol M.-Chir. J.*, 1913, xxxi. 268.

has continued to be worthily representative of the best in British Surgery. We congratulate the editors and publishers upon the brilliant success of their venture.

**Practical Prescribing.** By Arthur H. Prichard. Pp. xi, 307. London: Oxford Medical Publications. 1913. Price 6s. net.—This is a book put together on a somewhat original plan. The author has selected some thirty-five diseases, and details the treatment of each step by step. First he gives a short description of a case, then in parallel columns the treatment prescribed from time to time, and the objects for which each drug is exhibited. Then follow notes on the therapeutic action, indications and prescribing of the drugs used, and finally comments, not merely therapeutic, but more or less general in character. Thus the book reads like a series of clinical demonstrations, and as far as it goes will certainly be found of value to senior students. But the selection of diseases is a little arbitrary, and the detail so considerable that a very little goes a long way. The book can only be read with advantage in connection with the actual observation of cases; this is no disadvantage, as it renders it obviously unsuitable for "cramming." The treatment advised is generally on the orthodox textbook lines.

**The Practitioner's Practical Prescriber.** By D. M. Macdonald, M.D. Pp. 198. London: Oxford Medical Publications., 1913. Price 5s. net.—This little book seems a sort of hybrid between a miniature "Dictionary of Treatment" and the "Information" given in the first half of a well-known visiting list. First, there is an alphabetical list of morbid conditions, with a very brief description of each. Then comes the usual posological table, and lastly miscellaneous notes on prescribing, incompatibles, spas, sickroom cookery, and other things. In a chapter called "Therapeutic Brevities" we find notes such as this: "Phenacetin, most helpful in the bronchitis of children." Later on there is a table for calculating the duration of pregnancy. A form for drawing up a *post-mortem* report, though doubtless useful, is a somewhat grim addition to a book devoted to treatment.

**Materia Medica Notes.** By James A. Whitla. Pp. 157. Edinburgh: E. & S. Livingstone. 1913. Price 2s. 6d. net.—The author modestly disclaims the title of "Textbook" for this work, but for the average medical student it will be found to contain all that can properly be included under the title of materia medica in a concise form. There are also some pages of prescriptions and general notes on prescribing. The author is perhaps right in beginning with the drugs themselves, and afterwards giving an account of the official preparations. As in all books of this kind, the pharmacology and therapeutics are somewhat of the stereotyped order ; thus alcohol is described as a " stimulant," butyl chloral hydrate is said to " concentrate its action on the fifth nerve." Opium is said to be contra-indicated in kidney disease. Apart from this the book is carefully written and reliable, and would form a useful companion to such a work as Dixon's *Pharmacology*.

**Anæsthetics : their Uses and Administration.** By DUDLEY WILMOT BUXTON, M.D., B.S. Fifth Edition. Pp. xiv, 477. London : H. K. Lewis. 1914. 10s. 6d. net.—Those who are familiar with Dr. Dudley Buxton's *Anæsthetics* will welcome his new edition, and will find, as they might expect to find, that the work of revision has been carried out by the author with characteristic thoroughness, the whole book having been more or less re-written, and many additions having been made in both letterpress and illustrations. Excellent short descriptions have been included of intravenous and intratracheal ether, of dosimetric chloroform, and of spinal, regional and local analgesia. It would be impossible for any man to dogmatise as to the relative values of all the recently-introduced methods, and Dr. Buxton has adopted the course of stating the chief advantages claimed for them by their respective advocates, while at the same time he has drawn the reader's attention to the dangers and disadvantages which may be met with in their association. The chapter on local and regional analgesia now includes very useful plates taken from Rawling's well-known book on " Landmarks," and the subject has been afforded the prominence which the increasing popularity of these methods demands. The book is full of useful detail, but its size has been kept within reasonable bounds ; and its author is to be congratulated upon the masterly way in which he has coped with his task.

**Modern Anæsthetics.** By J. FREDERICK W. SILK, M.D. Pp. xii, 200. London : Edward Arnold. 1914. 3s. 6d. net.—By the title *Modern Anæsthetics* one might be led to expect a work dealing only with methods of recent invention or introduction, but Dr. Silk's book bearing this title is really a small textbook treating on all the established methods, old and new. As a consequence, room has not been found for more than short accounts of such subjects as intravenous ether, spinal analgesia, and intratracheal ether. The section on local and regional analgesia is clearly put, and should help to encourage the correct employment of drugs in this direction. On the whole, the work is eminently practical, and affords welcome and reliable guidance for students of this branch of medicine.

**The Sanitary Inspector's Handbook.** By ALBERT TAYLOR. Fifth Edition. Pp. xii, 612. London : H. K. Lewis. 1914. Price 6s. net.—This edition of this well-known work has been brought up to date and revised, especially as regards recent legislative enactments on public health matters. No detailed comment on its contents is necessary, beyond noting that the work of revision has been carried out in the same thorough and accurate manner which has made the previous issues standard books of reference for practical men.

**The Administrative Control of Small-Pox.** By W. McC. Wanklyn, D.P.H. Pp. vii, 86. London: Longmans, Green and Co. 1913. Price 3s. 6d net.—Dr. Wanklyn's large experience of small-pox gives this work a *prima facie* title to respect; his lucid and definite style makes the perusal of it easy and pleasant. His sound common sense, command of administrative detail, and practical organising ability as shown throughout its pages, make it a most helpful contribution to the literature of this important subject. It will be read, of course, with greatest advantage by those engaged in public health work, but we consider that medical men in general would be well advised in getting and reading it, especially when cases of small-pox occur in their vicinity. An intelligent appreciation of the work of the Medical Officer of Health, when he is faced by a possible outbreak of small-pox does not come by nature, as it were, even to trained clinicians and general practitioners, but is most desirable for them under the circumstances. We would even say that a certain section of the lay public, especially members of local councils and health committees, might perform their duties to the community more satisfactorily had they some knowledge of the matters very plainly set forth in this book. We hope it will be widely read, and its valuable teaching properly assimilated.

**Practical Nursing.** By the late Isla Stewart and Herbert E. Cuff, M.D., F.R.C.S. Fourth Edition. By H. E. Cuff and W. T. Gordon Pugh, M.D., B.S. Pp. x, 448. Edinburgh: William Blackwood & Sons. 1913. Price 5s. net.—A book which has reached its fourth edition, and has been reprinted eleven times, may be said to have stood the test of time, and established its reputation on a firm foundation. The present edition of this valuable work has been brought up to date, and will be found a most exhaustive and complete treatise, both on practical nursing and also on the general principles and scientific discoveries which underlie that art. The style is simple, clear and concise, and by the adoption of a smaller print the book has been kept within reasonable dimensions. Doctors as well as nurses may study its pages with advantage, especially those who cannot always depend on the services of highly-trained nurses for the care of their patients.

